# Supplementary material for: MLKL and CaMKII Are Involved in RIPK3-Mediated Smooth Muscle Cell Necroptosis
Source: Cells. 2021 Sep 12;10(9):2397. doi: 10.3390/cells10092397 (PMC8471540; doi:10.3390/cells10092397)
Supplement: Supplementary file 1 [file cells-10-02397-s001.zip › cells-1366639-supplementary.pdf]

Supplementary Figure S1

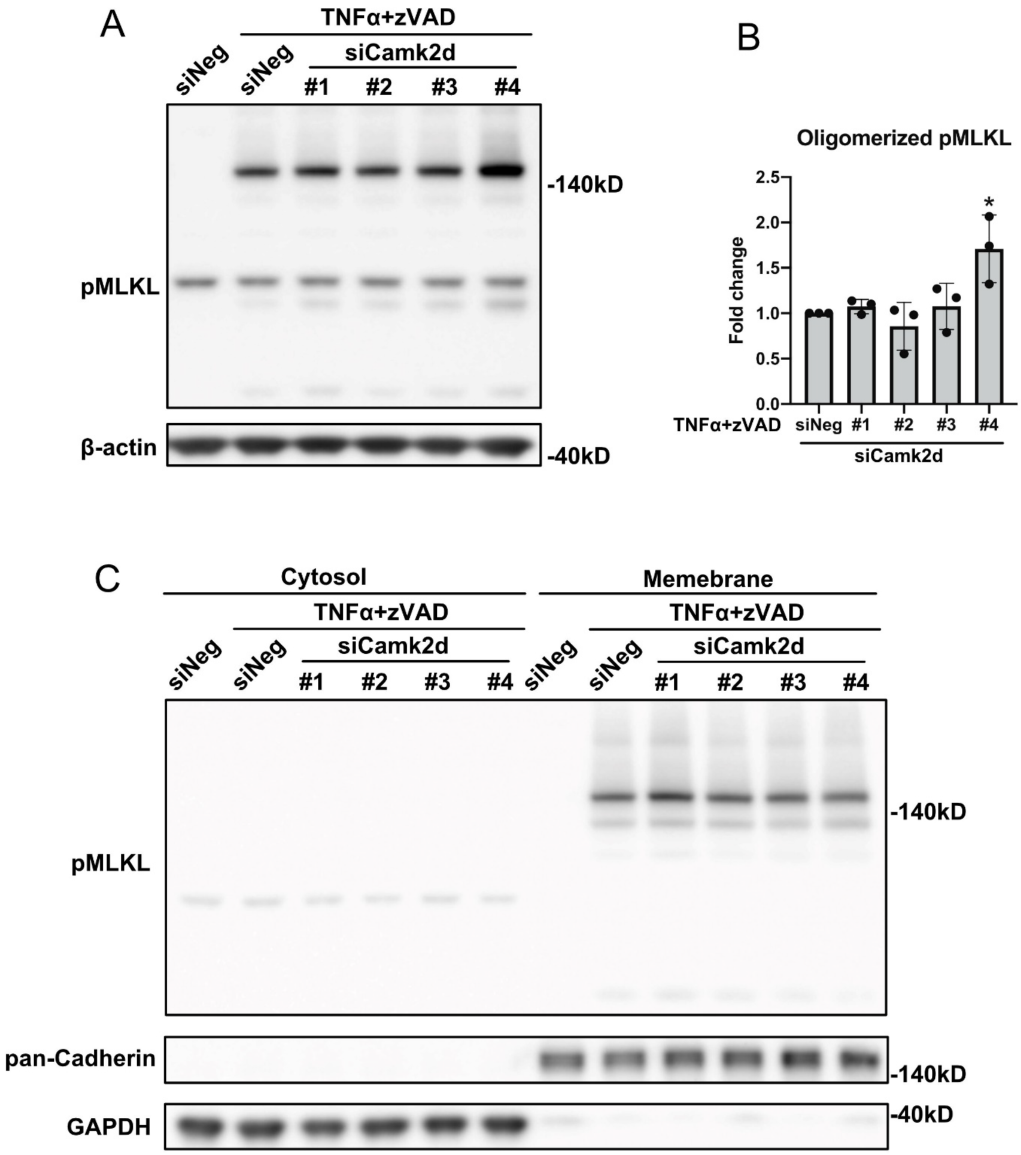

Supplementary Figure S1. Silencing CaMKII does not affect oligomerization or trafficking of phospho-MLKL. (A) MOVAS cells were transfected with siRNAs against *Camk2d* for 48 hours, cells were lysed in 1% digitonin buffer and subjected to Western blotting analysis with the indicated antibodies. **(B)** Quantification of (A). **(C)** Cytosol and membrane fractions were subjected to Western blotting analysis with the indicated antibodies. Data were presented as mean ± SD of at least three independent experiments. One-way ANOVA was performed in (B). *p<0.05 compared to TNFα plus zVAD treated group.

Supplementary Figure S2

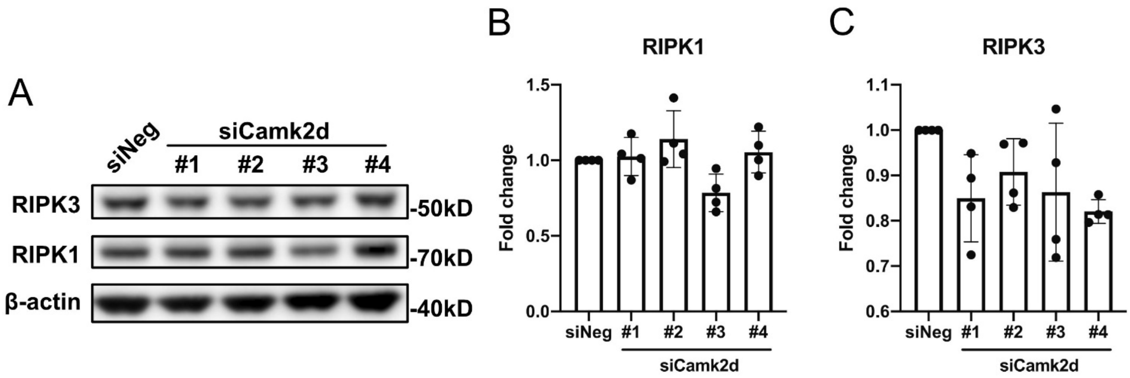

Supplementary Figure S2. Silencing CaMKII does not affect the levels of RIPK1 or RIPK3. (A) MOVAS cells were transfected with siRNAs against *Camk2d* for 48 hours, cells were lysed in RIPA buffer and subjected to Western blotting analysis with the indicated antibodies. **(B and C)** Quantification of (A). Data were presented as mean ± SD of at least three independent experiments. One-way ANOVA was performed in (B) and (C).
